# Viruses in marine sediments: a review of their effect on biogeochemistry and microbial interactions

**DOI:** 10.1128/aem.00275-25

**Published:** 2026-03-05

**Authors:** Jacob R. A. Williams, Jennifer F. Biddle

**Affiliations:** 1 School of Marine Science and Policy, University of Delaware 522742, Lewes, Delaware, USA; The Pennsylvania State University, University Park, Pennsylvania, USA

**Keywords:** virus, sediment, biogeochemistry

## Abstract

Viruses are known to impact the flow of carbon through the environment, while also impacting the microbial community around them. While this has been reexamined in recent years in the marine water column, viral impacts on marine sediments, the microbes, and carbon contained within are due for a reassessment. This review synthesizes findings from studies on marine sediment microbial communities to examine the extent of viral contribution to biogeochemical cycling, through ecological impacts as well as through cell lysis. Viruses have been shown to increase metabolic activity within the sediment microbial community as well as increase biodiversity, improving the range and ability of microbial communities to degrade organic matter. Viruses have also been found to have more direct effects on sedimentary geochemistry, with viral-mediated cell lysis allowing for the release of organic matter into the sediment while also being able to act as reservoirs for biologically relevant chemicals such as dissolved organic phosphorus. Viruses have been shown to impact the biogeochemistry of buried marine sediment, with less attention being paid to freshwater sediments and surficial marsh sediments. The interest in viral activities in sediments can help us to understand the drivers of biotic contributions to diagenesis.

## INTRODUCTION

In the ocean, viruses are known to mediate the carbon cycle, keeping dissolved organic carbon (DOC) in the microbial loop and preventing its movement into secondary consumers (1). However, few direct measurements of this carbon flux exist, and recent considerations of the ocean system consider that viruses can not only loop carbon in the dissolved pool but also participate in a carbon shuttle through the creation of aggregates that pellet or can be part of a viral vent that releases carbon into the atmosphere (2). While the pelagic ocean has been well reviewed for viral contributions to microbial diversity and biogeochemical cycling, less attention has been paid in recent years to the sedimentary portion of the ocean, where carbon is potentially stored over geological time, but viral influence can still be felt. Here, we review the literature on viruses in sedimentary ecosystems and their potential role in biogeochemistry and microbial diversity.

In sedimentary systems, microbes convert organic materials into forms of DOC (1). Within the sediment microbial community, DOC is cycled and recycled, where the growth of microbes on sunk organic material, their death, and their subsequent consumption by heterotrophs within the system can lead to the sequestration of organic matter within the benthos. This carbon may eventually be released in the form of inorganic or recalcitrant organic matter, which can either remain within the sediments or be released into the atmosphere or overlying water. In this sense, microbes control the amount of carbon burial or escape that occurs in the “blue carbon” economy of benthic marine systems (3). The processes within diagenesis are influenced by the environment, with temperature, oxygen availability, and other factors affecting the speed and efficiency of biological degradation (4). Less clear, however, is the role of viruses in the array of processes that dictate microbial degradation of organic matter, how microbial activity is regulated, and how viruses impact geochemical cycling within sedimentary environments.

Viruses can affect microbes through two main infection and replication pathways, distinguished by the method of replication and release. Lytic viruses propagate and lyse their host, whereas lysogenic viruses (also known as prophages or temperate viruses) integrate themselves into the bacterial genome, propagating during cell division (5). Of the two strategies, it has been found that lysogenic or temperate viruses are more prevalent than lytic viruses in these marine sediments (5, 6). Chronic bacteriophages, which integrate into the bacterial genome and release progeny without killing the host, have been characterized within the water column; however, characterization within the sediments remains a topic requiring study (7–9). Studies of viruses in sediments have often employed mesocosm studies, often with the addition of mitomycin C to induce viral expression (10). Studies rely on microscopy for enumeration and diversity observation; many utilize metagenomics to analyze diversity and metabolism, and geochemical techniques can reveal carbon and nutrient dynamics (10). Sediment samples must be dislodged from sediment particles and filtered in order to be enumerated via microscopy (10), which would not be an issue that occurs as commonly in water column viruses, as they would be floating within the water samples, but may still exist with flocculent layers of water (4). Although studies have shown that there are effects caused by these viral interactions, previous research on the association between viruses and burial of marine organic matter has been relatively sparse and inconclusive, particularly for shallow sediments. Viral lysis may significantly affect cycling of material through the microbial loop, potentially increasing the available organic matter for heterotrophs to consume. Given the nature of nitrogen and phosphorus as limiting factors for microbial degradation of organic matter (11), evidence of viral lysis improving the abundance of these nutrients may offer critical insight for further understanding of microbial degradation and sediment geochemical processes. Considering the importance of phosphorus for the degradation of organic compounds such as hydrocarbons (11), viral mediation of organic phosphorus levels may act as a key part in the rates of microbial degradation. Viruses are also able to contribute genes that can improve phosphorus metabolism, such as the *phoH* gene (12). This review examines the effect of viruses on marine sediment biogeochemistry by synthesizing findings that discuss the impact of viral infection on marine sediment communities in coastal, deep-sea, and hydrothermal vent ecosystems, with the ultimate goal to provide a comprehensive synthesis of the viral-microbial interactions within marine sediments and the broader implications that this has on global biogeochemical cycles.

## ENDEMISM AND IMPORTANCE OF VIRUSES IN THE SEDIMENT MICROBIAL COMMUNITY

Viruses are highly abundant in sedimentary systems, with around 10^7^−10^10^ viruses in 1 g of dry sediment (10, 13). Virus-to-bacteria ratios within marine sediments fluctuate around 10, lower than water column ratios (13). The origin of viruses in sediment is an area of debate. Evidence in support of endemism of viruses residing in sediments includes unique structures present in viruses and viral-microbial interactions (5, 6, 14). Sedimentary viruses bear traits that are rare in the water column, suggesting endemism of the viruses within the benthos (14). This information is corroborated by taxonomic analysis, where it was found that Siphophages differ in proportional abundance by 79% in sediments, compared to 50% in the water column (6). This would suggest, due to the differences between the two environments, that distinct viral taxa would thrive in their respective locations, and the higher abundance of sediment-bound viruses is due to their replication within the benthos. Metagenomic analysis reveals high levels of endemism within sediment systems, with several novel taxa of viruses observed within a range of environments such as seamounts, cold seeps, as well as trench slopes (15–18) Marine sediments also exhibit high viral diversity, with current models estimating that viral diversity is significantly higher in marine sediments as opposed to the water column (6, 15–17, 19). Consistent DOC with increasing depth further suggests viral endemism in sediments, as replication of viruses would lead to lysis of cells, releasing DOC (5). This endemism of viruses within marine sediments shows an importance in examining these communities and provides insight into considerations on the impact of these viruses on microbial communities.

## IMPACT OF VIRAL INFECTION ON ECOLOGY

Viral lysis within marine sediment microbial communities has a significant impact on several aspects of ecology, such as biodiversity, nutrient availability, metabolism, and sharing of genetic information (4, 5, 14, 20–27). Viral abundance is positively correlated with microbial abundance (12, 14, 28), highlighting their strong connection with the microbial community in ecological interactions. Biodiversity enrichment occurs as a result of viral infections under a “killing the winner” model (5), where dominant taxa that make up a majority of the microbes in the community are suppressed by cell lysis (4, 5). The suppression of abundant taxa allows for less dominant or competitive microbes to avoid competitive exclusion, increasing species richness. Lysis also plays a role in the provision of nutrients through the “viral shunt,” where organic material from lysed cells is released into the environment and returns to the microbial community, recycling microbially derived organic matter (5, 14, 20–26). The organic material generated from viral lysis provides nutrition to the microbial community, providing an approximate 2%–11% of organic nitrogen and phosphorus (21), leading to increased microbial activity (14, 20, 21, 23). Quantitatively, approximately 0.37–0.63 Gt carbon per year is exported to sediments via viral lysis (24), supplementing the nutrient-deficient benthos to allow for the growth and flourishing of microbes within both the shallow and deep sediment, with lysis in deep sediments maintaining a consistent amount of dissolved organic matter (DOM) in deeper sediment (5). Compared with the water column, where nutrient availability is a less-critical factor for the shaping of microbial communities, sediment microbial communities are shaped in diversity and provided nutrients to thrive by viral lysis (4, 14, 20, 21, 23).

Another effect of viral lysis on the marine sediment microbial community is horizontal gene transfer, which can occur through the production and subsequent integration of exogenous DNA (14, 29) or through direct gene transfer via lysogenic viruses adding genes to microbial genomes (27, 30). The release of exogenous DNA via lysis provides both a source of nitrogen and phosphorus as described above (14) and can also be absorbed and integrated into the genome of microbes (29, 31), allowing traits such as resistance to pathogens or ability to degrade and metabolize certain matter to be passed onto other organisms within the microbial community. On the other hand, lysogenic viruses integrate into the genome of microbes, providing genetic information which can include auxiliary metabolic genes (AMGs) to the host (27, 30). Within sediments of the Bohai Sea, Yellow Sea, and South China Sea, AMGs within viruses governing phosphate metabolism were found to make a large proportion of the viral community (12). The input of these AMGs can allow for host microbes to adapt to stresses in the environment (27). Additionally, high levels of novel genes in viruses (30) further highlight their integral role in marine sediments. The ability of viruses to drive gene transfer both through the release of exogenous DNA (14) and direct integration into the genome (27, 30) has implications for the ecological functions of marine sediment microbial communities, supporting the adaptation of the community to environmental changes and spreading traits across organisms.

## GEOCHEMICAL IMPLICATIONS FROM MICROBIAL INTERACTIONS

A variety of geochemical implications result from increased metabolic activity, diversity, and release of organic material from lysed cells (4, 5, 14, 20–26). Increased degradation of materials via microbes increases the release of dissolved inorganic carbon (14, 23) as well as more complex organic matter into sediments (5), leading to further sedimentation of sinking matter. Viral impact on geochemistry is further supported by the correlation between viral and microbial abundance (14, 22, 28), which implies that the presence of viruses may increase microbial abundance by this supplementation of more organic material. Given this increase in microbial abundance, biodegradation is likely improved by viral infection through increased microbial abundance and activity. Increases in the biodiversity of microbial communities assist in the degradation of organic material in higher quantities and variety, preparing it for further shifts in geochemistry, such as entrance into diagenesis or the release of inorganic carbon from the sediment (25).

Viral infection and interaction with the sediment microbial community through lysis and modification of gene expression also lead to increased metabolic activity of microbes (14, 20, 21, 23). Due to this increased microbial activity, organic matter is cycled through buried sediment faster, converting labile matter into longer-lasting, recalcitrant waste compounds to be further processed by other organisms. With viral abundance having a positive relationship with microbial activity, it is implied that viruses contribute to increased sequestration and degradation of materials in the marine sediments. The viral shunt also plays a role in this increased degradation, as the provision of nutrients via lysis (5, 14, 20–26) both improves metabolic activity and increases the sedimentation of organic matter from the water column and shallow sediments. The carbon export to the sediments from cell lysis (24) contributes to approximately 15%–30% of carbon released (25). Nitrogen and phosphorus, two nutrients serving as critical limiting factors in sediment microbial communities (11), are supplied via the release of exogenous DNA through viral lysis (22), increasing degradation of the organic matter within the benthos.

The assistance of viruses with horizontal gene transfer (27, 30) is another factor where viral impacts on ecology affect geochemistry. This is through the provision of AMGs, such as those governing SO_4_^2−^ reduction, which plays a role in the early diagenetic process of microbial degradation (27, 30). Viral lysis is known to improve sulfate reduction within microbial communities (14), further increasing its capability to break down organic matter. Lysogenic viral genomes also tend to contain metabolic genes for metabolizing complex polysaccharides, improving the ability of host cells to survive (15). Alongside other AMGs providing genes for dark carbon fixation and other metabolic functions, viruses provide a large amount of genetic information to hosts that improves the community’s ability to process organic and inorganic material (17). These ecological effects not only play a significant role in the shaping of marine sediment microbial communities but also in altering the geochemistry of these sediments.

## DIRECT EFFECTS OF VIRUSES ON SEDIMENTARY GEOCHEMISTRY

While the release of cellular material via lysis can lead to the export of nutrients to benthic microbial communities (24), the fate of this matter can vary between being further buried into the sediment (24) and being released back into the water column as CO_2_, which can then continue upward to be released into the atmosphere (25). Organic matter within marine sediment remains consistent in abundance with respect to depth (5) and is proportional to viral abundance (14, 23). This proportionality of viral abundance with organic matter abundance coupled with the improved microbial abundance and activity as a result of viral infection suggests that viruses may act as drivers for organic matter-degrading processes through viral lysis, providing matter to undergo diagenesis either through biotic processes (5, 14, 20–26) or abiotic processes such as temperature and pressure. It also shows that the organic matter seen within the sediment is not coming entirely from the water column, as there would have been a clear decrease in organic material with depth if this were the case. The transformation of carbon from lysed cells can then form more recalcitrant matter via diagenesis, increasing its longevity in sediments and resistance to being released back out of the benthos, potentially linking viral infection to increased longevity of carbon compounds in sediments. Looking into another important compound for the microbial community, phosphorus, viruses themselves are also suggested to be reservoirs for compounds such as dissolved organic phosphorus (DOP) (26), meaning that viruses may have the capacity of modulating the phosphorus content released from lysed cells, as well as potentially acting as a form of organic matter storage within the sediment. The ability of viruses to modulate and maintain organic matter within marine sediments acts as a driver for biogeochemical processing, as well as providing increased storage ability for compounds such as carbon through diagenesis and phosphorus, through viruses themselves likely acting as a storage for DOP (26). The evidence of viruses providing export of matter to the sediment (25) as well as releasing matter in the sediments themselves makes these phages an integral part of cycling for organic matter but may also play a role in carbon release.

**Fig 1:**
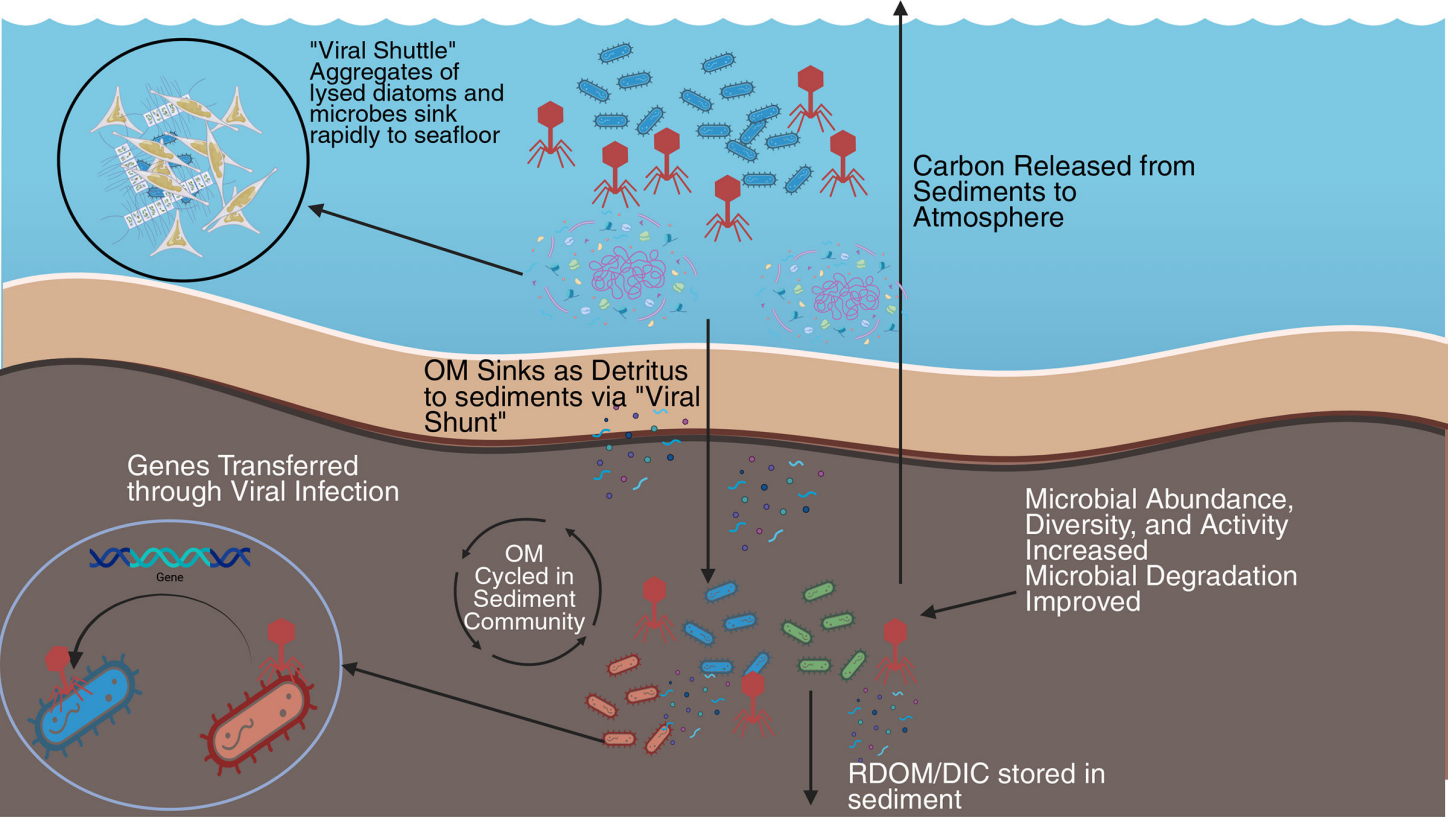
Diagram representing the impacts of viruses on aqueous sediment geochemistry. Organic matter (OM) sinks to the sediments, which is cycled in the microbial community, as well as increasing abundance, diversity, activity, and degradation of OM. From this “microbial loop” recalcitrant dissolved organic matter (RDOM) and dissolved inorganic carbon (DIC) are stored in sediments. Carbon is also released into the atmosphere from the sediment, and viral infection transfers genes between hosts or releases exogenous DNA to be used by bacteria, potentially aiding in metabolic activity. Created in BioRender. Williams, J. (2026) https://BioRender.com/b4wqimp.

On the other hand, viral lysis is observed to release carbon from the microbial community as well, with an estimated 0.37–0.63 Gt C yr^−1^ (25) being released from the ocean into the atmosphere as a result of viral interactions. This value is similar to the amount of carbon observed being exported to sediments (24), presenting evidence that viruses have a similar effect on the release of carbon from the ocean as they do in sedimentation. This process leads to a significant step in the carbon cycle, with inorganic and organic material being released from ocean sediment. Viral-mediated lysis of microbes increases both the burial within and release of organic matter from marine sediments. Release of DOC (5, 14, 23) and exogenous DNA (14) improves the ability for microbial communities to degrade matter. This degradation can lead to the long-term storage of recalcitrant matter, resistant to biodegradation and release from the sediment, or release from the sediment to the water, where it can eventually enter the atmosphere (Fig. 1) (25). The implications of this extend into applications of the “blue carbon economy,” as the release of carbon via lysis (25) could mark a reduction of expected efficiency, thus requiring further consideration and research.

## ENVIRONMENTAL VARIATIONS BETWEEN SHALLOW, DEEP, AND UNIQUE ECOSYSTEMS

Minor variations occur within different environments of marine sediments, with different factors taken into account and affecting the activity of microbial communities and viral infection. Shallow, coastal sediments show coupling of viral-microbial interactions with temperature, with increased activity observed with higher temperatures (23), a factor not considered in deep-sea sediments due to consistent low temperatures (5). Pressure, a factor which would be present in deep-sea sediments specifically, showed no effect on the activity of the local microbial community (24), and oxic conditions are observed within shallow sediments within both coastal and deep-sea environments (5, 14, 23). Despite the minor variations observed between the coastal and deep-sea environments, no significant effect on ecological and geochemical trends due to viral activity was observed.

Lysogenic viruses carry AMGs, which act as a major driver of horizontal gene transfer within hydrothermal vent ecosystems (27). Evidence for horizontal transfer of sulfate reduction genes was found within the sediment microbial community of hydrothermal vent systems (27), as well as the presence of a host of other novel genes (30). Hydrothermal vent ecosystems, having a high abundance of sulfur compounds in comparison to other marine sediments (27), mean that this gene transfer is of higher significance. Additionally, vent ecosystems exhibit a less diverse set of lysogenic viruses as opposed to other communities (21), showing high specificity for hosts within the vent system. As a result of the environmental conditions and high biodiversity of vent sediment ecosystems (27, 30), as well as the prevalence of gene transfer (27, 30), the hydrothermal vent provides a unique environment for viral-mediated geochemistry.

## UNKNOWNS IN VIRAL IMPACTS ON GEOCHEMISTRY

Despite the wealth of information regarding the effect of viruses on ecology and broader impacts on ecosystems, their effect on geochemical processes is still largely unknown. Of the studies on marine virus geochemistry, the focus is placed on the viral shuttle (2, 26) to either sink to the benthos (24) or the viral vent, released into the atmosphere (25). Studies have mostly focused on the ecological impact of viral infections, describing increased community diversity (4, 5) and metabolic activity (5, 14, 20–26), which can suggest geochemical impacts. However, direct effects on geochemistry remain limited to the release of organic matter from lysis (24, 25) or viruses acting as a reservoir themselves (26) within the marine realm.

Impacts on freshwater sediments are not as well understood, with a study inconclusive regarding impacts on carbon cycling (32). Furthermore, marshes, a location that has been considered quite important for global carbon sequestration strategies (3), remain understudied. The viral shuttle also needs more investigation, as viral impacts on diatoms can cause increased speed of burial and improved carbon export (33, 34). Aggregated materials may also be preferentially preserved in sediment (35), and the shuttle may present increased or different nutrient inputs to the sediment microbes. Anthropogenic impacts on the viriobenthos are also an avenue of study that warrants more consideration, as the loss of microbial biodiversity seen in sediments below fisheries (36) and the shift in community assemblages due to oil contamination (37) suggest significant changes in the ability of biodegradation. Given the rising importance of carbon sequestration as a method to counteract anthropogenic climate change, future research into viruses should cover their impacts on geochemistry, both in the potential increase of sequestration and in the shaping of the microbial community to aid in burial.

## SUMMARY AND OUTLOOK

Viruses play a significant role in the biogeochemical cycling of matter within marine sediment microbial communities. Viral infection is seen to positively affect the community through improving metabolic activity, increasing biodiversity, facilitating horizontal gene transfer, and providing nutrients, increasing the capability of microbial degradation within the marine benthic community (4, 5, 14, 20–27, 30). Viruses also directly input organic matter into the sediment via cell lysis (24, 25) and can act as a storage for compounds such as DOP (17). Variation between areas of shallow or deep-sea sediments remained minimal, save for considerations of temperature (23) in the coastal, shallow sediments and the heightened specificity of viruses within the hydrothermal vent sediment community (30). Anthropogenic impacts, such as organic matter input by fisheries or oil contamination, have been shown to impact the sediment community, with viral interactions and activity potentially being linked to contamination alongside shifts in geochemical cycling within the affected sediments (36, 37). Changing climate has been shown to impact viral loads in the environment (38). Despite what has been found, there still remains a great deal of information to find, such as elucidating these effects in freshwater environments, which remains inconclusive (32), as well as marshes, an area of consideration for carbon sequestration strategies (3).

Given the rising interest in carbon dioxide removal strategies, a more robust understanding of the ways in which viruses impact biogeochemistry is necessary. The gaps in knowledge regarding marsh sediments (3) as well as freshwater sediments (32) pose a large area in which further research could be incredibly useful, as these environments are more susceptible to anthropogenic effects due to proximity and use. With mounting efforts to utilize the carbon burial capability of marine sediments, it is also important to quantify storage times and turnover for carbon released via viral lysis to determine the efficiency of carbon burial compared to the potential release of carbon. The nature of virus-bacteria interactions and their effect on geochemical processes is still an area of study with a great deal to be found, and thus continued efforts in this field will not only aid in understanding an important part of geochemical cycles but also in understanding how anthropogenic activity will affect a field that is attempting to be used to counter the mounting issue of climate change.
